# Enzymatic Synthesis of Galactosylated Serine/Threonine Derivatives by β-Galactosidase from *Escherichia coli*

**DOI:** 10.3390/ijms160613714

**Published:** 2015-06-17

**Authors:** Sooyoun Seo, Joseph Rebehmed, Alexandre G. de Brevern, Salwa Karboune

**Affiliations:** 1 Department of Food Science and Agricultural Chemistry, McGill University, 21,111 Lakeshore, Ste Anne de Bellevue, QC H9X-3V9, Canada; E-Mail: sooyoun.seo@mail.mcgill.ca; 2 INSERM UMR_S 1134, Dynamique des Structures et Interactions des Macromolécules Biologiques (DSIMB), University Paris Diderot, Sorbonne Paris Cité, INTS, Laboratory of excellence, GR-Ex, 6, rue Alexandre Cabanel, 75739 Paris Cedex 15, France; E-Mails: joseph.rebehmed@univ-paris-diderot.fr (J.R.); alexandre.debrevern@univ-paris-diderot.fr (A.G.B.)

**Keywords:** β-galactosidase, transgalactosylation, serine/threonine derivatives, molecular docking, binding affinities, galactosyl acceptor

## Abstract

The transgalactosylations of serine/threonine derivatives were investigated using β-galactosidase from *Escherichia coli* as biocatalyst. Using *ortho*-nitrophenyl-β-d-galactoside as donor, the highest bioconversion yield of transgalactosylated *N*-carboxy benzyl l-serine benzyl ester (23.2%) was achieved in heptane:buffer medium (70:30), whereas with the lactose, the highest bioconversion yield (3.94%) was obtained in the buffer reaction system. The structures of most abundant galactosylated serine products were characterized by MS/MS. The molecular docking simulation revealed that the binding of serine/threonine derivatives to the enzyme’s active site was stronger (−4.6~−7.9 kcal/mol) than that of the natural acceptor, glucose, and mainly occurred through interactions with aromatic residues. For *N*-*tert*-butoxycarbonyl serine methyl ester (6.8%) and *N*-carboxybenzyl serine benzyl ester (3.4%), their binding affinities and the distances between their hydroxyl side chain and the 1′-OH group of galactose moiety were in good accordance with the quantified bioconversion yields. Despite its lower predicted bioconversion yield, the high experimental bioconversion yield obtained with *N*-carboxybenzyl serine methyl ester (23.2%) demonstrated the importance of the thermodynamically-driven nature of the transgalactosylation reaction.

## 1. Introduction

As the structural and molecular recognition roles of glycopeptides and glycoproteins in many biological systems are increasingly being recognized, the development of glycosylation strategies for the synthesis of these compounds has become of substantial importance in the fields of food, biology and pharmaceutical sciences. These glycoproteins/peptides can provide protection against proteolytic enzymes, cell–cell recognition, cell growth, and oncogenesis [1,2]. In addition, the improvements in the functional properties of carbohydrate conjugated food proteins, such as emulsifying activity, protein solubility, and thermal stability, have been previously demonstrated [3]. These advantages emphasize the need for a highly specific method for the formation of glycosidic bonds between carbohydrates and amino acids/peptides/proteins. Because of the presence of various functional hydroxyl groups in saccharides, sequential selective protection–deprotection steps of these functional groups are needed in order to control the stereochemical and regiochemical specificity of the glycosidic bond formed through chemical synthesis. Contrary to the chemical synthesis, enzymatic synthesis often offers regio- and stereospecificity to the glycosidic linkages [4].

**Figure 1:**
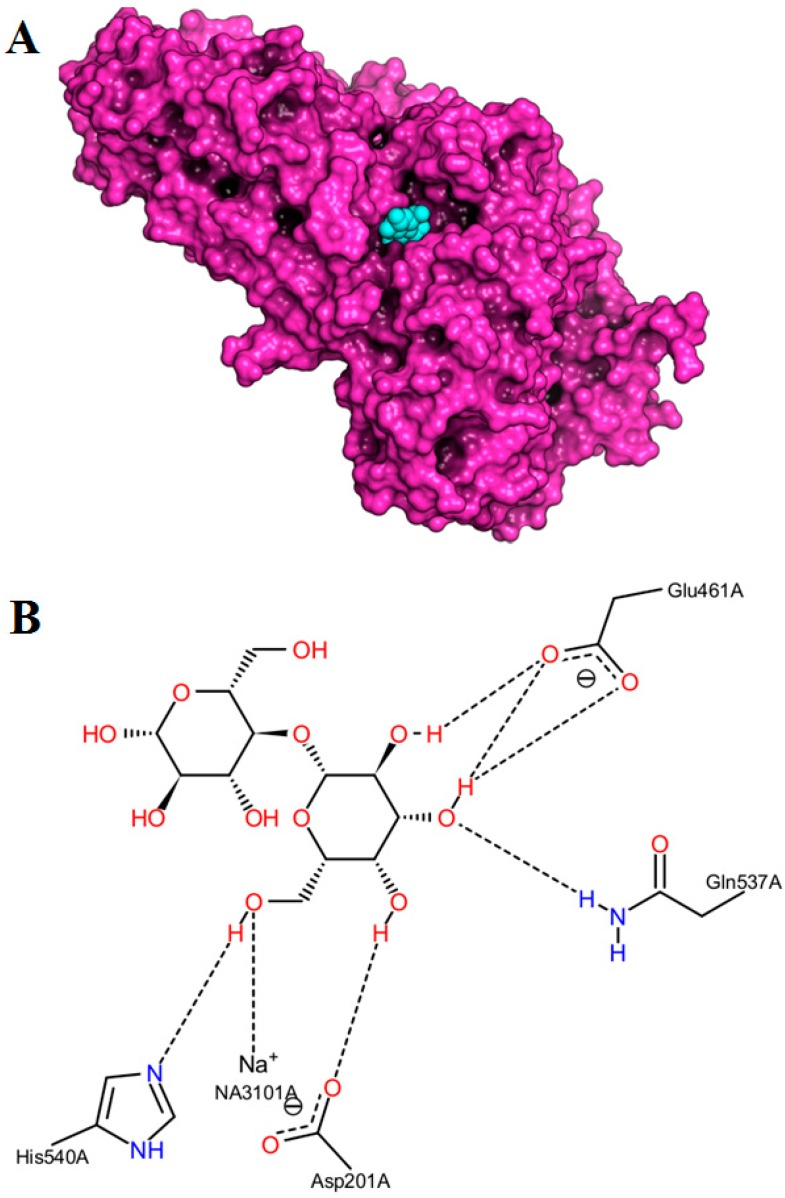
Experimental crystallography structure of the β-galactosidase lacZ of *E. coli* (Protein Data Bank code: 1JYN, chain A, surface, purple) (**A**) in complex with a lactose moiety (spheres, cyan) in the active site using PyMOL software; (**B**) Visualization of the interactions between the lactose moiety and the β-galactosidase enzyme with PoseView webserver.

β-Galactosidase (EC 3.2.1.23, Figure 1A) hydrolyzes the β-d-1-4 linkage of lactose releasing glucose and galactose as end-products or converting them into allolactose (β*-*d-1-6 linkage). This enzyme is a retaining glycosidase because it maintains the initial conformation of the anomeric carbon of the substrate [5,6,7]. The active site of β-galactosidase has two subsites with only one being highly specific for the galactose moiety. This lack of specificity of one subsite allows the binding of a wide variety of substrates, other than lactose, that can act as acceptors [5,6,7]. The first step of the mechanistic action of β*-*galactosidase-catalyzed reaction involves a cleavage of the glycosidic bond of the lactose or galactose-substituted molecules, and the formation of the covalent galactosyl-enzyme intermediate (Figure 1B) [7,8]. The second mechanistic step of β-galactosidase-catalyzed reaction involves the galactosyl transfer from nucleophile Glu537 to an acceptor. During this degalactosylation step, a transition state is formed and stabilized by interactions between Glu537, Try503, and the galactosyl ring oxygen. This transition state only forms in the presence of both incoming and leaving groups. The leaving group (glucose or *o*-nitrophenyl) can be replaced by a water molecule (hydrolysis) or by another acceptor substrate (transgalactosylation) [9]. The elucidation of binding affinity, interactions and orientations of selected substrates in the β-galactosidase’s active site can contribute to the understanding of its transgalactosylation efficiency and to the identification of a more suitable substrate for the effective production of galactosylated amino acid/peptide/protein derivatives. Only limited studies have investigated the galactosylation of amino acid/peptide by β-galactosidase [10,11,12,13,14,15,16]. None of these studies have used a structure-based computational approach to elucidate the binding of the amino acids at the galactosidase’s active site and to understand the effect of the amino acid’s blocking group on the synthesis of galactosylated amino acid derivatives. Structure-based computational methods, such as molecular docking, have been demonstrated to be useful in calculating the position and the orientation of a potential substrate in a binding site of an enzyme [17]. Most of the docking programs use empirical potential energy functions to calculate the binding energies of enzyme-substrate complexes, which involve van der Waals, Coulomb electrostatic interactions, and hydrogen bonds. In the present study, the syntheses of galactosylated serine/threonine derivatives through β-galactosidase lacZ from *Escherichia coli*-catalyzed transgalactosylation reaction were investigated. Molecular docking simulation was explored as a structure-based computational tool to apprehend the structural basis of the enzyme’s selectivity and to compare the relative binding affinities of the enzyme towards different serine/threonine derivatives.

## 2. Results and Discussion

### 2.1. Effect of Selected Reaction Media on the Transgalactosylation of Serine Derivative

The transgalactosylation of *N*-carboxy-benzyl-serine methyl ester (*N*-Z-Ser-OMe) by *E. coli* β-galactosidase using lactose or *o*-nitrophenyl β-d-galactopyranoside (ONPG) as donor substrate was carried out in buffer and in selected two biphasic reaction systems (Table 1). In order to reduce the water activity, 70.0% of organic solvent was used in the biphasic systems. Heptane (log *P* of 4.27, non-polar) and heptanone (log *P* of 1.98, mid-polar) were used as co-solvents in the biphasic reaction systems to investigate the effect of polarity of co-solvent on the transgalactosylation reaction. The hydrolyzing activity of *E. coli* β-galactosidase on ONPG decreased (<10.0%) in buffer and in selected biphasic reaction systems during the investigated time course (data not shown). Using ONPG as donor, the highest yield of transgalactosylated *N*-Z-Ser-OMe (23.2%) and the fastest initial rate (4680.0 μmol product/L.h) were achieved in heptane:buffer reaction system, whereas with the lactose, the highest yield (3.9%) and the fastest initial rate (1600.0 μmol product/L.h) were obtained in the buffer reaction system. These results are attributed to the higher solubility of ONPG in the biphasic reaction system as compared to the aqueous reaction media. The lower yields of transgalactosylated serine derivatives and the slower initial rate of the reaction obtained using lactose as donor in the biphasic reaction system (<0.1%‒0.6%, 40.0–200.0 μmol product/L.h) are likely due to the limited solubility of lactose in these reaction media. The higher yields of transgalactosylated *N*-Z-Ser-OMe obtained with ONPG were also reported by other authors [12]. Table 1 also shows that the yield of the transgalactosylated products was dependent on the type of organic solvent. As compared to heptane, the use of heptanone, denoted by its lower log *P* value as a co-solvent, resulted in lower yields (<0.1%‒0.2%) and slower initial rates (80.0–200.0 μmol product/L.h) indicating its detrimental effect on the enzyme’s transgalactosylating activity. These results are attributed to the fact that the hydrophilic solvents have higher tendency to strip off the water from the surface of the enzyme, which is essential for its catalytic activity [18,19].

The use of an excess of carbohydrate donor (ONPG or lactose) at molar ratios of 3:1 and 9:1 resulted in a decrease in the initial rate of the reaction and in the formation of galactooligosaccharides (data not shown). These results can be attributed to the high competition between *N*-Z-Ser-OMe and galactose in excess for binding the acceptor subsite of *E. coli* β-galactosidase, affecting the affinity of β-galactosidase-binding subsite for *N*-Z-Ser-OMe acceptor. The molar ratio of carbohydrate to serine derivative of 1:3 led to higher yields and to the fastest initial rate of the reaction. The excess of serine derivative seems to kinetically favor the transgalactosylation reaction over the hydrolytic one. Similarly, Holla *et al.* [14] have reported relatively high bioconversion yields (4.0%‒39.0%) of transgalactosylated products when using 2- to 3.5-fold higher concentrations of serine derivatives than ONPG. In contrast, Becker and Kuhl [12] have obtained higher yields in the presence of higher concentration of carbohydrates and attributed these results to their protective effect on the enzyme in the presence of organic solvents. The results also indicate that in the presence of lactose donor, no improvement in the transgalactosylation yield (<0.6%) was obtained at a molar ratio of carbohydrate to serine derivative of 1:3 or 3:1. In contrast, Layer and Fischer [16] reported that the *trans*-mono-galactosylation of serine with an excess of lactose yielded 28% of *N*-*tert*-butoxycarbonyl-1-*o*-β-d-galactopyranosyl-l-serine methyl ester. This high yield may be attributed to the use of lactose suspension and of serine with different protective group.

**Table 1:** Transgalactosylation of *N-*Z-Ser-OMe in aqueous and in aqueous-organic solvent reaction systems at selected substrate ratios.

Ratio of Sugar to Serine	Sugar	Reaction Time (h)	Bioconversion Yield (%) and Initial Rate of the Reaction (μmol/L.h) ^a^		
			Buffer	Heptane:Buffer (70:30)	Heptanone:Buffer (70:30)
1:1	ONPG	5	<0.1	2.0	0.1
		12	0.3 (0.01) ^b^	0.4	<0.1
		24	<0.1	<0.1	<0.1
	Lactose	5	3.9 (0.40) ^b^	<0.1	0.2 (0.05) ^b^
		12	0.5	<0.1	0.1
		24	<0.1	<0.1	<0.1
1:3	ONPG	5	<0.1	3.9 (1.17) ^b^	<0.1
		12	<0.1	23.2	<0.1
		24	<0.1	19.2	<0.1
	Lactose	5	<0.1	0.1 (0.01) ^b^	<0.1
		12	<0.1	0.6	<0.1
		24	0.1	<0.1	<0.1
3:1	ONPG	5	<0.1	3.8 (0.40) ^b^	0.2 (0.02) ^b^
		12	<0.1	2.9	<0.1
		24	<0.1	<0.1	<0.1
	Lactose	5	<0.1	<0.1	<0.1
		12	<0.1	<0.1	<0.1
		24	<0.1	<0.1	<0.1
9:1	ONPG	5	<0.1	0.9 (0.09) ^b^	<0.1
		12	<0.1	<0.1	<0.1
		24	<0.1	0.8	<0.1
	Lactose	5	0.2 (0.02) ^b^	0.3 (0.03) ^b^	<0.1
		12	0.1	<0.1	<0.1
		24	<0.1	<0.1	<0.1

^a^ Experimental results are averages of triplicates. Standard deviations were less than 10%; ^b^ Initial rate of the reaction (μmol product/L.h) in parenthesis.

### 2.2. Transgalactosylation of Serine/Threonine Derivatives

**Figure 2:**
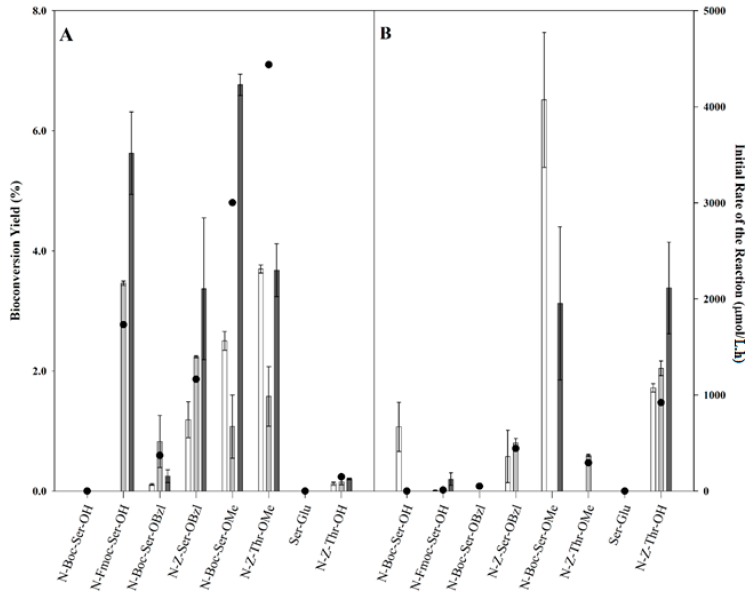
Transgalactosylation yields (left axis) of serine/threonine derivatives by *E. coli* β-galactosidase in heptane:buffer (70:30) using (**A**) ONPG, or (**B**) lactose as donors at 5 h (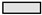), 12 h (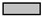), and 24 h (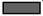) of reaction and the initial rates of the reaction (right axis, ●).

It has been demonstrated that the selection of the amino blocking group is important for the improvement of the bioconversion yield of transgalactosylated amino acid derivatives [14]. Selected serine/threonine derivatives with different protecting groups were investigated as acceptors and their effects on the transgalactosylating efficiency of *E. coli* β-galactosidase were assessed using ONPG (Figure 2A) or lactose (Figure 2B) as substrate donor. The identified optimal reaction conditions, including heptane:buffer mixture (70:30) and 1:3 sugar to serine derivative molar ratio, were used. Compared to *N*-Z-Ser-OMe (23.2%), the transgalactosylation of the investigated serine/threonine derivatives exhibited lower yields (Table 1); however the use of *N*-*tert*-butoxy-carbonyl l-serine methyl ester (*N-*Boc-Ser-OMe) as acceptor resulted in a faster initial rate of the reaction (7816.8 μmol/L.h) with lactose as donor as compared to *N*-Z-Ser-OMe (4680.0 μmol/L.h) demonstrating the higher affinity of the *N-*Boc-Ser-OMe acceptor towards the enzyme and the possibility of obtaining higher yields. Different results were obtained by Cantacuzene *et al.* [20] who reported lower yield for the transgalactosylation of *N*-Z-Ser-OMe (8.00%) by β-galactosidase, but higher one with *N-*Boc-Ser-OMe (15.00%). These differences in the bioconversion yields reveal the significant effects of the reaction media, the availability of the substrates and the kinetic behavior of the reaction. Indeed, the produced transgalactosylated products, β-d-galactopyranosyl-l-serine/threonine derivatives, may be hydrolysed by *E. coli* β-galactosidase. Therefore, the bioconversion yields obtained reflect a balance between the formation and the hydrolysis of these products, whereas the initial reaction rate shows the rate of the product formation before the hydrolysis becomes predominant. This is demonstrated in our results by the decrease in the bioconversion yields within the investigated time course such as in the reaction with *N-*Boc-Ser-OMe where the minimum bioconversion yield was obtained at 12 h. The results also show that, although *N-*Fluorenyl-methyloxy-carbonyl l-serine (Fmoc-Ser-OH) led to the third highest bioconversion yield of transgalactosylated serine derivatives using ONPG as donor (5.62%, Figure 2A), the initial rate of the reaction was slower (1731.2 μmol/L.h) than the initial rate obtained using *N*-carboxy-benzyl l-threonine methyl ester (*N*-Z-Thr-OMe, 1.11 μmol/L.h). Both threonine derivatives with the same carboxy-benzyl (Z) amino blocking group, *N*-Z-Thr-OMe and *N*-carboxy-benzyloxy l-threonine (*N*-Z-Thr-OH), gave comparable bioconversion yields of 3.70% and of 3.38% using ONPG and lactose as donors, respectively; however, a faster initial rate could be obtained when using ONPG as donor for *N*-Z-Thr-OMe (4437.2 μmol/L.h) as compared to using lactose as donor for *N*-Z-Thr-OH (920.4 μmol/L.h). These results are due to the differences in the hydrophobicity between the two threonine derivatives. *N*-Z-Thr-OMe being more soluble in the organic phase, its transgalactosylation was more favored in the presence of hydrophobic ONPG.

No transgalactosylated products were obtained in the reaction containing the dipeptide serine-glutamic acid (Ser-Glu) with no protection groups. This result indicates the importance of protecting groups in the transgalactosylation reaction-catalyzed by *E. coli* β-galactosidase as emphasized by others [13,14,20]. In addition, the significant difference between the yields and the initial rates obtained using acceptors with the same *N*-protecting groups suggests that the *C*-protecting groups may play an important role in the transgalactosylation of the acceptor. Indeed, the acceptors with the same *N*-Boc protecting groups had significantly different yields and different initial rates depending on the *C*-protecting group: 0.8%/370.8 μmol/L.h (-OBzl, ONPG), and 6.8%/3001.2 μmol/L.h (-OMe, ONPG). The acceptors with the same *N-*Z protecting groups also had significantly different yields and different initial rates depending on the *C*-protecting group: 3.4%/1163.6 μmol/L.h (-OBzl, ONPG), and 23.2%/4680.0 μmol/L.h (-OMe, ONPG). This difference is likely due to the relatively bigger size of the -OBzl protecting group of the serine derivative that limits the access to the active site, as compared to the derivative with -OMe group. The yields and the initial rates also differed depending on the donor substrates, ONPG or lactose, suggesting the significant effects of acceptor/donor interactions and of the substrate availability in the enzyme’s microenvironment.

**Figure 3:**
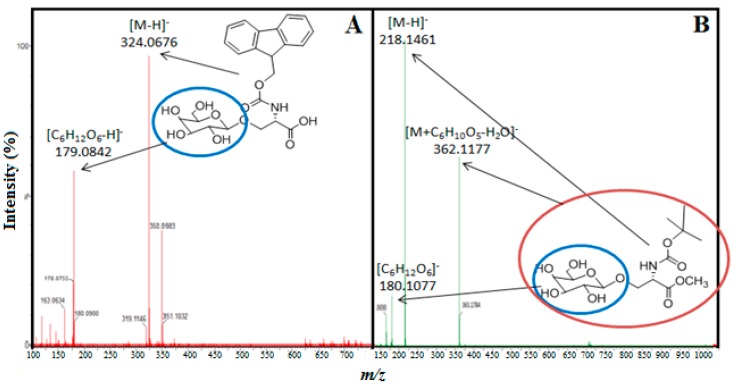
MS/MS spectra of (**A**) fragmented transgalactosylated *N*-Fmoc-Ser-OH and (**B**) fragmented transgalactosylated *N*-Boc-Ser-OMe.

The reactions in which the acceptors gave higher bioconversion yields of transgalactosylated products were analyzed by MS/MS to confirm the product formation. Only stronger signals of galactosylated product ions, galactosylated *N*-Fmoc-Ser-OH (Figure 3A) and galactosylated *N*-Boc-Ser-OMe (Figure 3B), were fragmented in tandem mass spectrometry. The numerous fragmentation reactions on the sugar moiety producing many ions at relatively low intensities can lead to difficulties in the identification of the fragments [21]. The fragmentation pattern mostly constituted of the ions with a complete loss of the sugar moiety from the serine derivative. The galactosylated *N*-Fmoc-Ser-OH at *m*/*z* of 490.5 underwent fragmentation, leading to abundant molecular acceptor ions [M − H]^−^ (324.1 *m*/*z*), galactose (179.1 *m*/*z*), and other fragments. The galactosylated *N*-Boc-Ser-OMe at *m*/*z* 400.4 underwent fragmentation leading to abundant molecular acceptor ions [M − H]^−^ (218.1 *m*/*z*), galactose (180.1 *m*/*z*), and unfragmented product ions [M + C_6_H_10_O_5_ − H_2_O]^−^ (362.1 *m*/*z*) and other fragments.

### 2.3. Docking Simulations

**Figure 4:**
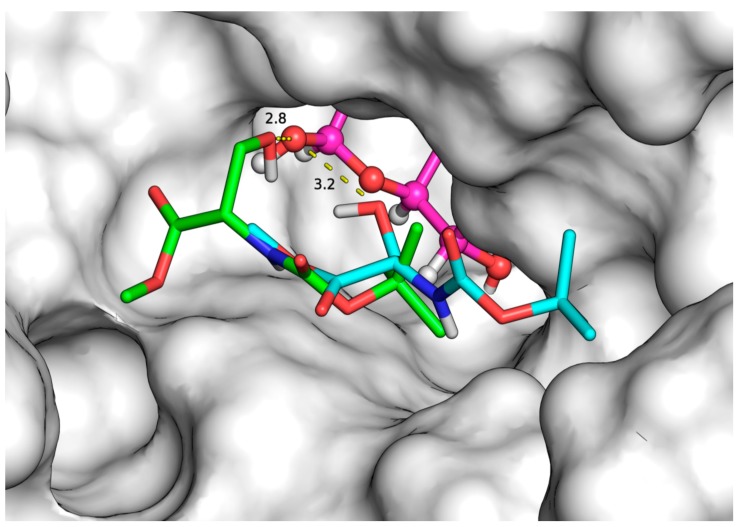
*N*-Boc-Ser-OMe acceptor substrate bound to β-galactosidase according to two of the three possible binding modes, carbon atoms are colored in green and cyan, oxygen atoms in red, nitrogen atom in blue galactose moiety in pink, hydrogen bond in dashed yellow line, and enzyme surface in white.

**Figure 5:**
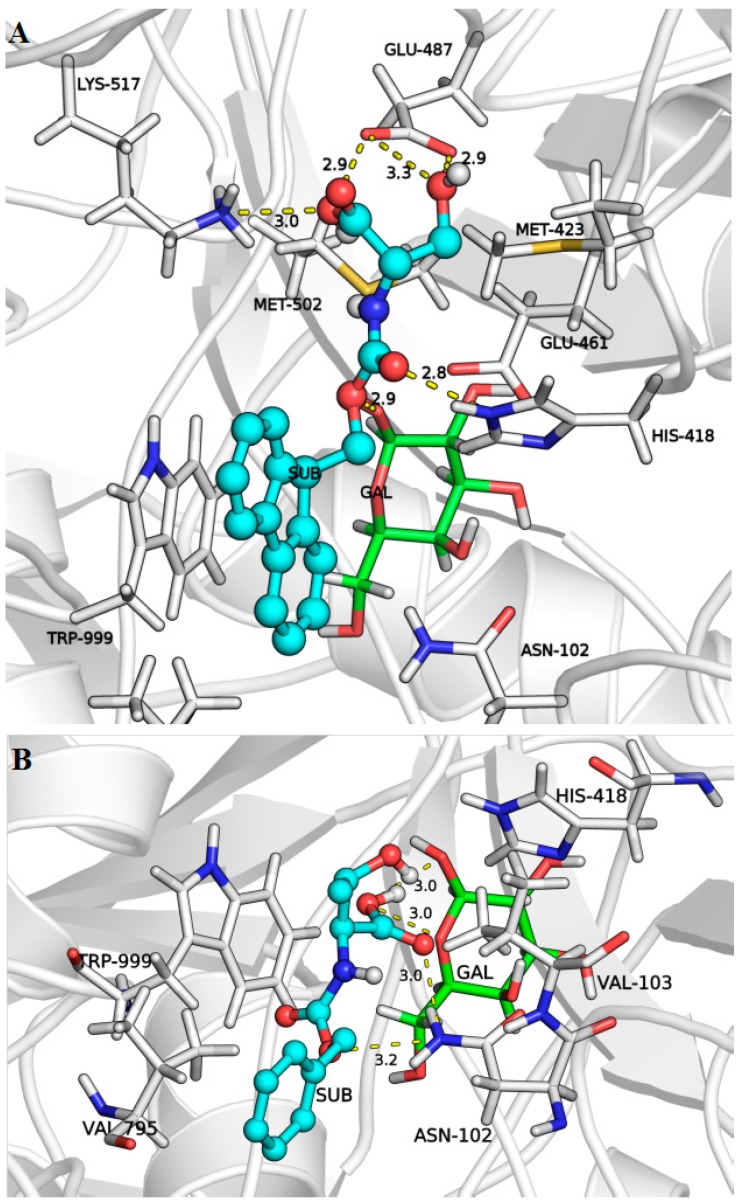
β-galactosidase enzyme in predicted complex with galactose moiety and (**A**) *N*-Fmoc-Ser-OH or (**B**) *N*-Z-Thr-OH. Enzyme is shown in cartoon presentation with the residues at the active site in sticks; galactose (sticks, green); *N*-Fmoc-Ser-OH/*N*-Z-Thr-OH (ball and sticks, cyan), hydrogen bonds (dashed lines, yellow); carbon (green and cyan), oxygen (red), sulfur (yellow), and nitrogen (blue) atoms.

In order to elucidate the possible orientation and the molecular binding of the acceptor substrates to the second subsite of lacZ β*-*galactosidase from *E. coli* during the second mechanistic step of the transgalactosylation reaction, molecular docking simulations were performed. The available crystallographic structure of galactose-enzyme complex in deep binding mode (2-*F*-α-d-glycosyl enzyme intermediate, 1JZ2) [8] was used as the enzyme model for the docking analysis. It has been demonstrated that the substrate of the reaction needs to move toward the deeper mode of binding, which is accompanied by a conformational change of the enzyme before transgalactosylation can occur [8]. The interaction between the enzyme and the galactose moiety occurs mainly via hydrogen bonds, with the equatorial OH groups of galactose [8]. During the docking simulations, each acceptor was positioned at the active site of the enzyme using twenty different orientations, and special attention was given to the binding modes that led to the strongest affinities and to the closest distances between the hydroxyl group of the acceptor and the 1ʹ-OH of the galactose moiety (Table 2). For instance, Figure 4 depicts two of the three possible docking solutions of *N-*Boc-Ser-OMe. Similarly to the leaving group of the natural substrate, glucose moiety of lactose [8], it was observed that most acceptor substrates formed π–π stacking interactions with Trp999 (Figure 5A,B). Other possible identified interactions include hydrogen bonding with the surrounding residues such as the carboxylic acids of Glu487, and the amino group of Lys517, and His418 (Figure 5A).

**Table 2:** Predicted binding affinities of the acceptors and the distance between the hydroxyl group of the serine/threonine derivatives and the 1′-OH of galactose moiety predicted by docking calculations at a specific binding mode of the acceptor.

Substrates (Acceptors)	Initial Rate (μmol/L.h)	Binding Modes	Binding Affinity (kcal/mol)	Distance (Å)
*N*-Fmoc-Ser-OH	1731.2 ^a^	1	−7.1	3.7
		2	−6.3	2.8
*N*-Boc-Ser-OBzl	370.8 ^a^	1	−6.2	3.1
		2	−6.0	3.0
		3	−5.9	2.9
		4	−5.7	3.1
		5	−5.5	3.0
*N*-Z-Ser-OBzl	1163.6 ^a^	1	−7.0	3.0
		2	−6.6	2.7
		3	−6.6	2.8
*N*-Z-Ser-OMe	4680.0 ^a^	1	−4.9	3.1
*N*-Boc-Ser-OMe	7816.8 ^b^	1	−4.7	2.8
		2	−4.6	3.2
		3	−4.5	3.0
*N*-Z-Thr-OMe	4437.2 ^a^	1	−5.7	2.7
		2	−5.5	2.9
*N*-Z-Thr-OH	920.4 ^b^	1	−5.7	3.6

^a^ ONPG as substrate; ^b^ Lactose as substrate.

The best-identified docking solutions that can favor the synthesis of transgalactosylated product were reported (Table 2) and correlated with the experimental results (Figure 2). The overall results show that the binding affinities of the serine/threonine derivatives (−4.5~−7.1 kcal/mol) in the second subsite of β-galactosidase were similar or stronger than those predicted for glucose (−4.4~−5.1 kcal/mol) and galactose (−4.1~−5.1 kcal/mol) as acceptors (data not shown). This may be due to the larger size of serine/threonine derivatives leading to better interactions with the amino acid residues found in the large acceptor-binding site of the enzyme as compared to the smaller monosaccharides. However, these binding affinities were weaker than the experimental binding affinities of ONPG (−8.1~−8.2 kcal/mol) at the degalactosylation step [22,23]. This slightly weaker binding affinity of *E. coli* β-galactosidase towards its acceptors may also reflect the importance of the thermodynamic equilibrium between the transgalactosylation and hydrolysis reaction. If the binding of the substrate at the subsite were both specific and tight, then the transgalactosylation reaction would be kinetically predominant. Conversely, if the binding were very weak, then the hydrolysis reaction would dominate [9].

The results (Table 2) show that the binding mode (#1) identified for *N-*Fmoc-Ser-OH leading to the highest binding affinity (−7.1 kcal/mol) did not allow its hydroxyl group to be closer (3.7 Å) to the 1ʹ-OH of the galactose moiety positioned in the first subsite. As compared to *N-*Fmoc-Ser-OH, the binding mode (#1) of *N*-Boc-Ser-OMe exhibited low affinity (−4.7 kcal/mol), but it favored the interaction between the hydroxyl group and the 1ʹ-OH of the galactose, which were closer (2.87 Å). Indeed, closer distance values (<3.5 Å) are expected to favor the formation of the glycosidic linkage between the galactose moiety and the serine/threonine derivatives. The docked solutions can explain the slower initial reaction rate obtained with *N*-Fmoc-Ser-OH (1731.2 μmol/L.h) acceptor as compared to *N-*Boc-Ser-OMe (7816.8 μmol/L.h). On the other hand, *N-*Z-Ser-OBzl acceptor exhibited relatively higher binding affinities (−6.6~−7.0 kcal/mol) and closer distances (2.7–3.0 Å) at the best-identified binding modes, but led to moderate initial reaction rate (1163.6 μmol/L.h); these results can be explained by the larger size of its *C*-protecting group, which may have sterically prevented its displacement into the deeper part of the enzyme’s active site. Similarly, *N-*Boc-Ser-OBzl with the same *C*-protecting group led to the slowest initial reaction rate (370.8 μmol/L.h), despite its high binding affinities (−5.5~−6.2 kcal/mol) and the closer distances between its hydroxyl group and the 1′-OH of galactose moiety (2.9–3.1 Å). As compared to *N*-Boc-Ser-OBzl, the fastest initial reaction rate was obtained with the acceptor substrate *N-*Boc-Ser-OMe (7816.8 μmol/L.h) with the same *N*-protecting group but with a smaller *C*-protecting group, although it exhibited lower binding affinities (−4.5~−4.7 kcal/mol). For the substrate *N-*Z-Thr-OH, the lack of a protecting group at its carboxylic end led the latter to interact with the 1ʹ-OH group of the galactosyl moiety instead of the hydroxyl group of the threonine as shown in Figure 5B leading to slower initial reaction rate (920.4 μmol/L.h) as compared to *N*-Z-Thr-OMe (4437.2 μmol/L.h). Only one binding mode was found for *N-*Z-Ser-OMe where its side chain hydroxyl group was favorably placed in relation to the 1′-OH of the galactose moiety. As compared to other serine/threonine derivatives, the lower binding affinity (−4.9 kcal/mol) and the relatively higher estimated distance (3.1 Å) for *N*-Z-Ser-OMe were not in accordance with its fast initial rate of the reaction (4680.0 μmol/L.h) in heptane:buffer media (23.2%). The combination of the experimental and *in silico* results highlights the importance of the acceptors/solvent interactions and reveals the thermodynamically driven nature of the investigated transgalactosylation reaction. To better understand the effect of substrate/solvent interactions, molecular dynamic simulations can be used. However, Brás *et al.* [24] have demonstrated that due to the lower selectivity of the second subsite of *E. coli* β-galactosidase and the lower binding affinity of the acceptors in this subsite, the initial configurations of the acceptor molecules found through docking are maintained during their molecular dynamics simulations. In contrast, using molecular dynamic simulations, Pérez-Sánchez *et al.* [25] have found a better orientation of the substrate, *N-*acetylglucosamine, in the active site upon a favorable substrate-solvent interaction although no differences in the protein flexibility and in the positioning of the active site residues of *E. coli* β-galactosidase were found in glycerol-derived solvents as compared to water.

## 3. Experimental Section

### 3.1. Materials

The enzyme, *E. coli* β-galactosidase (purified and stabilized with phosphate buffer salts), lactose, ONPG, the serine and threonine derivatives and other chemicals were purchased from Sigma (St. Louis, MO, USA).

### 3.2. Enzymatic Transgalactosylation

Prior to each enzymatic reaction, stock solutions of lactose or ONPG and *N*-carboxy-benzyl-serine methyl ester (*N*-Z-Ser-OMe) were prepared in 50 mM sodium phosphate buffer pH 7.8 with 2 mM magnesium chloride. These stocks solutions were mixed with the appropriate amount of heptane and heptanone to achieve substrate molar ratios of 1:1, 1:3, 3:1, 9:1 (1 = 50 μmol) in heptane:buffer and heptanone:buffer (70:30) reaction mixture media. To initiate the reaction, β-galactosidase (80 U/mL) was added to the reaction mixture. All reactions were performed in an incubator at 40 °C and with continuous shaking at 200 rpm. Transgalactosylation reactions were run in triplicate alongside controls containing no enzyme or no substrate and were monitored at specific time intervals over the course of 24 h of reaction. Initial rates of the reactions were extracted from the time progression curves. Aliquots of reaction mixtures were dried off under vacuum, using an Automatic Environmental Speed Vac system (Savant Instruments Inc., Holbrook, NY, USA).

### 3.3. Acceptor Specificity

The acceptor specificity of *E. coli* β-galactosidase was investigated using serine and threonine derivatives as acceptors including *N-*Boc-Ser-OMe, *N-*Boc-Ser-OH, *N-*Fmoc-Ser-OH, *N*-Z-Thr-OMe, *N*-Z-Thr-OH, *N-*Boc-Ser-OBzl, *N*-Z-Ser-OBzl, and Ser-Glu. The transgalactosylation reactions were run as described previously using a substrate molar ratio of 1:3 in heptane: sodium phosphate buffer reaction mixture (70:30).

### 3.4. Analytical Methods

The quantification of galactosylated serine/threonine derivatives was performed using a Waters HPLC system (Milford, MA, USA) equipped with TSK Gel-Amide 80 column (4.6 × 250 mm, 5 μm particle size, TOSOH Bioscience LLC, Montgomeryville, PA, USA) equipped with a UV diode-array detector (Model 2998) and a refractive index detector (Model 410). The elution of the reaction mixture was conducted with acetonitrile:water mobile phase (82:18) at a flow rate of 0.7 mL/min. Amount of galactosylated serine/threonine derivatives was estimated using Breeze software (Waters). The peaks of the products were identified by comparing the UV absorption spectra of the serine/threonine derivatives and by identifying a correlated change in refractive index due to galactosylation. The bioconversion yield (%) was calculated as the amount of galactosylated serine/threonine derivatives over the initial amount of acceptors.

Accurate mass measurements and MS/MS analyses for the identification of produced transgalactosylated serine/threonine derivatives were performed using a Synapt G2-S instrument (Waters) in positive and in negative electrospray and resolution modes. Only the results from the negative electrospray ionization have been presented. MS/MS spectra were acquired on defined masses with a 30 V collision energy.

### 3.5. Computational Analysis

The experimental structures of β-galactosidase from *E. coli* in complex with a lactose moiety (PBD ID: 1JYN) or a galactose moiety (PDB ID: 1JZ7, resolution 1.5 Å) [8] were used as model for Figure 1 and for the docking simulations, respectively. Autodock Vina search method, which consists of a genetic algorithm combined with local gradient optimization, [26] was used to generate 20 different binding modes (docked poses) for each ligand with a grid box of 25 × 25 × 25 Å^3^ positioned at the active site of the enzyme. A geometric criterion was used, *i.e.*, the OH group of amino acid close to 1ʹ-OH of the galactose moiety to form the linkage during the transgalactosylation. Then, the most favorable binding modes according to predicted binding affinities were selected and analyzed. Visualization of the 3D enzyme/substrates complexes were made using PyMOL (The PyMOL Molecular Visualization System, Version 1.6, Shrödinger, LLC, Portland, OR, USA). The 2D complex diagram was produced by PoseView webserver [27].
